# Exploring Psychosocial Burdens of Diabetes in Pregnancy and the Feasibility of Technology-Based Support: Qualitative Study

**DOI:** 10.2196/53854

**Published:** 2025-04-21

**Authors:** Maya V Roytman, Layna Lu, Elizabeth Soyemi, Karolina Leziak, Charlotte M Niznik, Lynn M Yee

**Affiliations:** 1 Division of Maternal-Fetal Medicine Department of Obstetrics and Gynecology Northwestern University Feinberg School of Medicine Chicago, IL United States; 2 Stritch School of Medicine Loyola University Chicago Maywood, IL United States; 3 Brown University Providence, RI United States; 4 University of Michigan Medical School Ann Arbor, MI United States

**Keywords:** digital health, mHealth, pregnancy, psychosocial, social determinants, technology, diabetes, burdens, qualitative analysis, mobile apps, feasibility

## Abstract

**Background:**

Gestational diabetes mellitus and type 2 diabetes mellitus impose psychosocial burdens on pregnant individuals. As there is less evidence about the experience and management of psychosocial burdens of diabetes mellitus during pregnancy, we sought to identify these psychosocial burdens and understand how a novel smartphone app may alleviate them. The app was designed to provide supportive, educational, motivational, and logistical support content, delivered through interactive messages.

**Objective:**

The study aimed to analyze the qualitative data generated in a feasibility randomized controlled trial of a novel mobile app designed to promote self-management skills, motivate healthy behaviors, and inform low-income pregnant individuals with diabetes.

**Methods:**

Individuals receiving routine clinical care at a single, large academic medical center in Chicago, Illinois were randomized to use of the SweetMama app (n=30) or usual care (n=10) from diagnosis of diabetes until 6 weeks post partum. All individuals completed exit interviews at delivery about their experience of having diabetes during pregnancy. Interviews were guided by a semistructured interview guide and were conducted by a single interviewer extensively trained in empathic, culturally sensitive qualitative interviewing of pregnant and postpartum people. SweetMama users were also queried about their perspectives on the app. Interview data were audio-recorded and professionally transcribed. Data were analyzed by 2 researchers independently using grounded theory constant comparative techniques.

**Results:**

Of the 40 participants, the majority had gestational diabetes mellitus (n=25, 63%), publicly funded prenatal care (n=33, 83%), and identified as non-Hispanic Black (n=25, 63%) or Hispanic (n=14, 35%). Participants identified multiple psychosocial burdens, including challenges taking action, negative affectivity regarding diagnosis, diet guilt, difficulties managing other responsibilities, and reluctance to use insulin. External factors, such as taking care of children or navigating the COVID-19 pandemic, affected participant self-perception and motivation to adhere to clinical recommendations. SweetMama participants largely agreed that the use of the app helped mitigate these burdens by enhancing self-efficacy, capitalizing on external motivation, validating efforts, maintaining medical nutrition therapy, extending clinical care, and building a sense of community. Participants expressed that SweetMama supported the goals they established with their clinical team and helped them harness motivating factors for self-care.

**Conclusions:**

Psychosocial burdens of diabetes during pregnancy present challenges with diabetes self-management. Mobile health support may be an effective tool to provide motivation, behavioral cues, and access to educational and social network resources to alleviate psychosocial burdens during pregnancy. Future incorporation of machine learning and language processing models in the app may provide further personalization of recommendations and education for individuals with DM during pregnancy.

**Trial Registration:**

ClinicalTrials.gov NCT03240874; https://clinicaltrials.gov/study/NCT03240874

## Introduction

Gestational diabetes mellitus (GDM), defined by the World Health Organization as a condition of hyperglycemia in pregnancy with blood glucose measurements exceeding normal values but below values diagnostic of diabetes, and type 2 diabetes mellitus (T2DM) during pregnancy impose greater burdens and correlate with greater health risks including increased rates of maternal and neonatal morbidity than diabetes mellitus (DM) outside of pregnancy [1]. Antenatal DM management mitigates these risks [2]. Current DM intervention methods in the United States focus primarily on medical therapies, such as medication regimens, blood glucose maintenance, and medical nutrition therapy (MNT) [2]. Although evidence is clear that improved glycemic control during pregnancy results in improved perinatal outcomes, ample data suggest that social and structural determinants of health can preclude full achievement of DM goals in this critical life period [3-5].

However, there is less evidence about the experience and management of potential psychosocial burdens of DM during pregnancy. Psychosocial factors related to DM diagnoses encompass the environmental, social, behavioral, and emotional elements that can affect long-term care. These factors, which include diabetes-related distress (ie, treatment burden and fear of uncertain future health outcomes), economic distress, and mental well-being challenges can impact adequate provision of self-care and adherence to medical treatment plans [6]. For example, pregnant individuals with DM display more depressive symptoms, with prenatal stress and depression serving as a predictor for postpartum stress and depression [7,8]. In addition, the intersectionality of psychosocial stress with other social determinants of health, such as poverty and limited education, may compound the challenges of being pregnant with DM. For instance, Muhwava et al [9] identified greater levels of anxiety and stress in low-income pregnant women with GDM in South Africa and Yee et al [3] qualitatively characterized how the interplay of socioeconomic, psychological, and epistemic factors serves as a barrier to DM self-management during pregnancy. Psychosocial consequences of navigating GDM or diabetes in pregnancy have been characterized in the literature. A systematic review on women’s experiences with a diagnosis of GDM found several qualitative studies revealing that individuals felt isolation, abandonment, and guilt after an initial GDM diagnosis, along with frustration with GDM management and financial burden of following treatment regimens [10-12]. Similar themes of diabetes-related distress, fear, and exhaustion were observed in recent studies examining the experiences of individuals with pre-existing diabetes in pregnancy [13,14]. Identifying potential psychosocial burdens, as a part of a multilevel understanding of social determinants of DM-related health during pregnancy, is thus crucial in the management of pregnant people with DM, as it may influence not only their perinatal outcomes, but long-term health outcomes as well.

Given this need for a comprehensive resource for individuals with DM in pregnancy, our team developed a novel mobile app for education and mitigating DM. Digital health technology, such as health-related smartphone apps, has proliferated in recent years in clinical, research, and public consumer spheres to help individuals with DM adjust to lifestyle interventions and maintain health care engagement [15]. SweetMama, a smartphone app leveraging the potential of digital health technology to improve health outcomes, was created to support DM management among low-income pregnant people [16]. In this secondary analysis of data from SweetMama participants, we aimed to identify the psychosocial burdens of having DM during pregnancy and sought to understand how a smartphone app may help alleviate such burdens.

## Methods

### Overview

This qualitative study evaluated data collected during a feasibility randomized controlled trial conducted among low-income, English-speaking pregnant people aged 18 years or older, who had a diagnosis of GDM or T2DM. Individuals with type 1 DM were not eligible for inclusion, given their greater use of continuous glucose monitors and insulin pumps. In this feasibility trial, individuals were randomized to use the SweetMama app (n=30) or usual care (n=10) from the diagnosis of DM during pregnancy through 6 weeks post partum. Unbalanced randomization was intentional since this is a feasibility study, and it was desirable to have greater exposure of the participants to SweetMama use. Results from the primary trial are currently under analysis. Participants received routine clinical care at a single, large academic medical center in Chicago, Illinois.

The SweetMama app was designed to provide supportive, educational, motivational, and logistical support content, delivered through interactive messages via the app, tailored to participants’ treatment, reminders for appointments, and featured a library of trusted contents, that were received 3 times a week. This educational content included recipes, documents from clinicians, trusted educational websites, clinic information, SweetMama instructional videos, and videos on how to take insulin and safely dispose of needles. Participants also set goals for their DM management with their clinical team and would receive customized reminders weekly in the SweetMama app to monitor their progress in achieving their goals. SweetMama was not designed as a glucose-logging app, but rather served to provide diverse, low health literacy, user-friendly education and tips, alongside a repository of recipes, local resources, and trustworthy educational material. Further details about SweetMama have been previously described [17,18].

All participants completed in-depth, qualitative exit interviews after delivery about their experience of having DM during pregnancy. SweetMama users were also queried about their perspectives on the app. Interviews were guided by a semistructured interview guide and were conducted by a single interviewer [JJ; refer Acknowledgments section] with extensive training in empathic, culturally sensitive qualitative interviewing of pregnant and postpartum people. The interviewer identified as a Black woman researcher. The interview guide was designed by the investigator team based on extensive previous experience providing clinical care for and interviewing pregnant people on these topics, including during earlier usability phases of investigation before the development of this version of SweetMama [16-19]. The interview guide also adapted concepts from the Technology Acceptance Model, and it was cognitively tested on nonparticipants before its deployment in the study [20]. Interviews were approximately 40 to 60 minutes in length and were conducted in-person in a private location. These interviews were audio-recorded and professionally transcribed verbatim.

Interview data were analyzed using a grounded theory constant comparative technique [21,22]. This method of thematic analysis consists of line-by-line readings of interview transcripts which are coded for concept patterns, termed as subthemes, and then collapsed as overarching themes through review and discussion by research coders. Two researchers [LL and ES] conducted this thematic analysis of interview content to identify emergent themes related to the psychosocial burdens of living with DM during pregnancy and the potential role of the app in mitigating those burdens, if any.

### Ethical Considerations

Written consent was provided by all participants, and this study was approved by the Northwestern University institutional review board (IRB study number: STU00205409). The original consent form allowed for secondary analysis of participant data without additional consent. Study data were deidentified for researchers conducting the qualitative thematic analysis of the interview transcripts. Participants received compensation in the form of gift cards (US $100) for their participation in the trial.

## Results

### Overview

From January 14, 2020, to October 10, 2020, a total of 40 participants enrolled in the parent randomized controlled trial, of whom 30 participants were randomized to use SweetMama. Of the 30 SweetMama users, 18 had GDM and 12 had T2DM. Majority of those participants identified as non-Hispanic Black or African American (n=17, 57%) or Hispanic (n=12, 40%). Most SweetMama users (n=21, 70%) had completed at least some college or technical school education. All 40 participants met study criteria for low-income status, and a majority (n=33, 83%) had publicly funded prenatal care (Table 1).

**Table 1:** SweetMama participant demographics.

Variable of interest and groups			Usual care (n=10)		SweetMama (n=30)
Age (years), mean (SD)			31.3 (6.9)		31.4 (4.7)
**Race and ethnicity, n (%)**					
	Non-Hispanic Black or African American	8 (80)		17 (56.7)	
	Hispanic	2 (20)		12 (40)	
	Non-Hispanic White	0 (0)		1 (3.3)	
Medicaid or Medicare, n (%)			8 (80)		25 (83.3)
**Education, n (%)**					
	Some high school or less	0 (0)		3 (10)	
	High school graduate	5 (50)		6 (20)	
	Some college or technical school	3 (30)		9 (30)	
	College or technical school graduate	2 (20)		12 (40)	
**Relationship status, n (%)**					
	Married	3 (30)		14 (46.7)	
	Single	5 (50)		7 (23.3)	
	Other	2 (20)		9 (30)	
Nulliparous, n (%)			2 (20)		6 (20)
**Current work status, n (%)**					
	Work full-time	5 (50)		11 (36.7)	
	Work part-time	1 (10)		8 (26.7)	
	Unemployed	2 (20)		4 (13.3)	
	Other	2 (20)		7 (23.3)	
BMI at first prenatal visit (kg/m^2^), mean (SD)			36.7 (9)		42.9 (12.4)
**Diabetes diagnosis, n (%)**					
	GDM^a^	7 (70)		18 (60)	
	T2DM^b^	3 (30)		12 (40)	

^a^GDM: gestational diabetes mellitus.

^b^T2DM: type 2 diabetes mellitus.

Thematic analysis of participant interview data focused on the psychosocial burdens of DM during pregnancy identified 5 major themes, namely challenges of taking action, negative affectivity regarding diagnosis, diet guilt, difficulties managing other roles and responsibilities, and reluctance to use insulin (Table 2). In addition, our analysis yielded findings on how the SweetMama smartphone app helped mitigate these burdens in each of these domains. The themes regarding mobile health (mHealth) support mechanisms included: enhancing self-efficacy, capitalizing on external motivation, validating user efforts, maintaining medical nutrition therapy, extending clinical care, and building a sense of community (Table 3). Each theme, with exemplary quotations, is discussed below.

**Table 2:** Psychosocial burdens of diabetes diagnosis during pregnancy.

Theme	Exemplary quotation
Challenges of taking action	“Well, like I knew what I needed to do… The challenge is actually doing it and going about it. So not so much of the knowledge part. More of the action part to me.”
Negative affectivity regarding diagnosis	“I was not even like, because like I said at first I was in no way, shape or form okay with being gestational diabetes, ya know?”
Diet guilt	“You know, sometimes it’s hard...you have to be really strong willed. Because if you’re, not that I ate bad or I had an unhealthy diet, but...whenever I feel like I’m told you can’t do something that’s when you...most want it. So that’s like the most difficult part...I found. It’s just hard staying away from food.”
Difficulty managing other roles and responsibilities	“I’m pregnant, I’m a full-time student...it was a little bit overwhelming to be able to maybe put a little bit more energy...due to COVID and just not being able to really go out to the stores...the way I typically would, that kinda discouraged me because it's like I wasn’t really able to handle stuff the way I usually would outside. It’s like as soon as I start using the app and stuff, what was going on with the virus and then you know stores were closed. It was just a lot going on at the time.”
Reluctance to use insulin	“I said I didn’t want to be on insulin. I didn’t want to take the medication at first, so that’s how it was.”

**Table 3:** Role of a smartphone app in mitigating psychosocial burdens during pregnancy.

Theme	Exemplary quotation
Enhancing self-efficacy	“Probably the goals part again because when you’re at like a certain level, like for example if you have diabetes and now you can do something about it, so if you see where you’re at and where you need to reach that would motivate action.”
Capitalizing on external motivation	“Having more of a…even though you’re doing this but this is what’s helping you and this is what’s helping your baby, a little bit more education on that track, it would make a new mother feel a little bit more secure in what they’re doing.”
Validating user efforts	“Interviewer: So what you’re saying is you know what to do but it’s good to have that reminder. Why do you feel that way?” “Interviewee: Because it feels like you’re doing right, I don’t know. It’s good to have some confirmation because I didn’t have this before and sometimes I would think like or whether or not like I can eat something or I can go for a walk now or like after.”
Maintaining medical nutrition therapy	“I really want to adapt a better diet for myself. I know that, you know I can do it, because I’ve done it in the past, so I feel like that app will help me incorporate different things...eating the same thing can be boring, so the recipe portion of it was wonderful. Just seeing different things you can create out of stuff you already have in the house, as opposed to having to go buy expensive diet food all the time.”
Extending clinical care	“[Seeing videos of my providers in the app] showed that they cared. And to give patients more feedback on their health...It was like you took them home with you.”
Building sense of community	“I feel like it would be like a community of people to kinda come together as far as especially for people like me that never had diabetes before, to be a little bit more like educational help…with that process.”

### Psychosocial Burden Characterization

The first burden theme was the challenges of taking action*.* When asked about their experience with goal setting, participants reported struggling to act on the expectations to maintain their DM. Participants specified that they did not have difficulty understanding their goals, but rather they struggled with their motivation to be proactive and achieve their goals.

Second, negativity affectivity regarding diagnosis, in which participants experienced forms of denial of their diagnosis, emerged as a psychosocial burden, particularly for individuals with gestational, rather than pregestational, DM. Participants noted that they did not fully come to terms with their DM diagnosis:

I was really hesitant toward the beginning…like you know we just can’t control our bodies and it’s just a hormonal change.

Others described having a highly negative affect toward the diagnosis, which hindered their ability to fully engage in DM-focused self-care, such as taking medication and following MNT.

Next, many individuals reported experiencing diet guilt*.* Participants commonly reported that they struggled with transitioning their diets and described feeling guilty when they were nonadherent to recommendations. Participants noted they often wanted to “give up” by consuming food outside of their prescribed MNT plan:

I really got the taste for ice cream because I’m pregnant, my hormones are telling me ice cream.

They specified that recommended foods “may not have been what [they] exactly wanted to eat.” When participants “gave in” to their cravings for nonrecommended foods, participants experienced guilt, decreasing their motivation to continue with MNT. This cyclic effect of frustration and defeat was reported by several to be a challenge regardless of when their DM was diagnosed.

Fourth, participants described the difficulty of managing other roles and responsibilities. Participants felt overwhelmed by external circumstances, such as taking care of their children or the COVID-19 pandemic, which overlapped with this trial. Oftentimes, because of these circumstances, participants did not have time to or were unable to care for themselves. These responsibilities hindered participants’ abilities to receive care and optimally manage their DM. Furthermore, participants demonstrated insight into the ways in which these competing priorities limited their ability to fully engage in care and expressed guilt for not being able to fully participate in recommended health care.

The final psychosocial burden theme was reluctance to take insulin*.* Participants reported hesitancy taking prescribed insulin due to uncertainty about how it will affect their health and their fetus:

To be honest I didn’t want to [take insulin]...I don’t know why I felt like when I had my daughter it was the reason she was colicky.

This reluctance was often based on fear and uncertainty. Although participants did not typically disagree with medical recommendations, they did acknowledge concerns over what was unknown to them.

### mHealth Strategies to Reduce Psychosocial Burdens

SweetMama users offered feedback on the potential role of mHealth in alleviating the psychosocial burdens of DM in pregnancy (Table 3). Themes in this domain focused on how their use of the SweetMama app reduced the stresses of having DM, provided motivation, or validated behaviors.

The first facilitating theme was regarding enhancing self-efficacy*.* Participants reported that SweetMama empowered them to take action to manage their DM. SweetMama helped participants form beliefs that they were in control and had a choice in their DM treatment. Features such as recipes and access to resources helped maintain participants’ sense of agency:

…if you need more information, you can click on that and they’ll tell you more about what the subject was.

Participants noted that as actions appeared easier to complete with the support of the tool, and that as tasks became easier, they were more motivated to complete them:

the simpler the recipes and the simpler the exercises…the easier things like you could apply it into your life, then the more likely the person will be willing to do it.

Next, capitalizing on external motivation emerged as a benefit of SweetMama. Participants noted that SweetMama helped them harness their motivating factors, such as the health of their baby, to maintain their DM. For some participants, particularly when not intrinsically motivated for their own health, the attentiveness to the long-term goal of neonatal health was more motivating than the concept of promoting maternal health. When feeling a lack of motivation for self-care, participants were reminded that the health of their body reflected the health of their baby.

The theme validating user efforts was commonly endorsed. Participants felt SweetMama reassured them in their actions in managing DM, encouraging them to continue their efforts. This self-regulatory theme demonstrated the importance of feedback, even when it was not personalized; SweetMama, for example, did not provide customized feedback, but the generic supportive messaging and reassurance was seen as sufficiently beneficial. In addition, specific input from SweetMama, such as the reminders and messages tailored to treatment regimen and gestational age, provided validation to participants to quell uncertainties in their approach to DM management.

The fourth theme supporting the use of SweetMama was regarding maintaining medical nutrition therapy*.* Users identified that SweetMama aided in the maintenance of MNT by providing educational information, recipe ideas, and visual support. For some participants, the information provided by SweetMama on how to adopt a DM-focused diet during pregnancy helped them see the MNT goals as achievable rather than overwhelming and rigid. Participants favored the recipes on SweetMama to navigate their new diets. Participants especially noted that the variety of recipes allotted them creative freedom with food choices, fostering greater enthusiasm.

Fifth, SweetMama was lauded for its role in extending clinical care. Participants expressed that SweetMama added onto and supported the goals participants established with their clinical team. Unlike stand-alone apps that are unrelated to the health care team, SweetMama engaged the patient and her care team together in the process of goal setting. Even though SweetMama intentionally did not contain a direct patient-provider communication portal, the representation of the clinical team in the content helped participants feel connected to their clinical team, as participants were able to access support even when not physically with their medical providers.

Finally, participants noted that SweetMama served a therapeutic role by building a sense of community*.* SweetMama was effective in helping participants feel part of a community and did not have to navigate DM alone. Specific features in the educational curriculum, such as messages and reminders, dispelled feelings of isolation and worries.

## Discussion

### Principal Findings

It has become widely understood that pregnancy serves as a window to improving long-term health [23]. Addressing challenges of DM during pregnancy may not only improve pregnancy outcomes but may also support long-term health benefits and health care engagement. We observed that psychosocial burdens present many challenges for DM self-management during pregnancy, particularly for low-income individuals who may experience greater challenges accessing resources. Here, we identified the use of a patient-centered mHealth tool like SweetMama is perceived by participants to effectively provide motivation, behavioral cues, and access to educational and social resources to alleviate these multilevel burdens of having DM during pregnancy. These broader benefits of SweetMama were previously explored in earlier developmental work as well [17].

A diagnosis of DM in pregnancy has been characterized in literature to spur a multitude of emotions, such as failure, despair, uncertainty, and fear [9,24]. This emotional complexity and depth were validated by our study with participant themes relating to guilt, challenges to action, and difficulty with managing responsibilities. These identified burdens are supported in a recent study conducted with Medicaid-enrolled pregnant individuals with T2DM, in which Fareed et al [25] identified themes among patient semistructured interviews, such as difficulty in “managing exhaustion” and “adherence to a new regimen,” where fatigue and hardships in adopting lifestyle changes may exacerbate chronic conditions, like diabetes. Future work is required to understand if these emotional responses to diagnosis and management DM in pregnancy are associated with adverse perinatal outcomes and whether existing mental health issues undergird an increased risk to DM in pregnancy, potentially due to intersecting social, structural, and environmental influences [26]. Regardless, it is important to support individuals and prevent negative experiences during pregnancy, even in the absence of such causal relationships. Thus, understanding tools to alleviate these psychosocial burdens are critical to providing “whole person” care.

Regarding alleviation of psychosocial burdens in this study, participants reported the SweetMama intervention enhanced self-efficacy, capitalized on external motivation, provided positive reinforcement to maintain behaviors, extended clinical care, and built a sense of community. Giving specific attention to self-efficacy as an important quality of maintaining one’s health with independence, we acknowledge that there are limited studies on DM management self-efficacy [27]. Self-efficacy is an important determinant in the success of and adherence to DM management. Increased self-efficacy may impact adherence to healthy behaviors, and it has been suggested that web-based curricula can also successfully increase DM management self-efficacy [27,28]. For instance, participants in a web-based education reported greater understanding of the elements of healthy eating and implementation of exercise [28]. However, this curriculum solely focused on managing aspects such as healthy lifestyle and diet. For this reason, we desired to examine and create a holistic, comprehensive curriculum that includes diet recommendations as well as appointment reminders, motivational messages, and other educational elements that inform DM self-management. Participants in this study stated a perceived enhancement of feeling empowered through the educational components of SweetMama, which in conjunction with the goal-oriented and appointment reminders, can facilitate adherence and engagement. Furthermore, previous literature examining GDM and mental health in minoritized populations has demonstrated increased risk of adverse mental health outcomes among individuals with GDM due to individual and structural burdens [29]. SweetMama, in contrast to other lifestyle interventions for people with GDM [30], has unique advantages as a comprehensive and accessible resource by touching upon components of GDM education, clinical engagement, and community building in 1 central hub. Furthermore, a qualitative meta-synthesis, which includes the initial SweetMama usability testing data, suggests that mHealth interventions are useful as a personalized and supportive tool for behavior change [31]. SweetMama’s use of provider videos in its content library and user-friendly curricular messaging allowed for a more personal experience with the detailed educational content in contrast to similar apps that were also supportive in GDM and DM management in pregnancy but had added issues with technical usability which led to frustration [32].

### Strengths and Limitations

Strengths of this study include its in-depth, patient-centered focus on the patient experience, as well as its purposeful inclusion of an understudied population [17]. In addition, given that this study was performed among a multicultural sample, having an interviewer with training in culturally sensitive qualitative interviewing was an advantage to ensure conversations between the interviewer and participants were empathic and nonjudgemental. Limitations of the study include that the study’s participants were from a single urban medical center with the majority identifying as non-Hispanic Black, limiting generalizability to other geographic regions or other types of health care settings. Second, since only English-speaking patients were included in the study, compounding burdens related to language and health literacy accessibility with a DM diagnosis may have been missed in the thematic analysis. A third limitation of the study is that the data presented are a secondary analysis of the primary SweetMama feasibility trial. Therefore, interview questions regarding psychosocial burdens of having diabetes in pregnancy were not directly probed, but were elicited by participants in their general feedback about their pregnancy experience and perceptions from using SweetMama. Fourth, as for any qualitative study, there is potential for bias during the process of constructing common thematic codes for unique and distinct responses from individual participants. Nonetheless, having 2 independent thematic coders as this study did helps to minimize individual research biases. Future work for SweetMama development includes greater linguistic and cultural adaptation for accessibility to a wider audience. Data from the primary feasibility trial of SweetMama are forthcoming, which will help elucidate whether a mHealth technology intervention such as SweetMama can improve perinatal outcomes among individuals with DM. Should the primary trial evidence support the clinical use of SweetMama, future work may include understanding how to integrate its use into health systems or individual clinics to promote the psychosocial well-being of pregnant people with diabetes.

### Comparison to Previous Work

Various mHealth technologies for individuals with diabetes in pregnancy have been assessed in the literature. These apps, such as MobiGuide for gestational diabetes by Peleg et al [33], Pregnant*+* by Borgen et al [34]*,* and Pears by Kennelly et al [35], focus predominantly on biometric data collection, with only a portion of the app dedicated to personalized goal setting and education [36]. Our findings from this secondary analysis, similar to previous studies investigating behavior change and self-management of DM in pregnancy, support that mHealth technologies provide an avenue for individuals to engage in personalized educational content aligned with clinical recommendations [37]. Studies described how pregnant individuals wished to obtain personalized recipes and exercise customization through mHealth apps [37,38], which are features that were already integrated in SweetMama and well received by participants. In addition, a similar app called “MySweetGestation,” developed by Tumminia et al [39], was designed for women who have diabetes or are at risk of developing diabetes during pregnancy and also used interactive, app-based engagement strategies with the user to provide personalized information and monitoring, though it also did not incorporate a repository of recipe suggestions nor evaluated potential effects of using the app on psychosocial burdens of experiencing DM during pregnancy. As seen in our study, participant satisfaction with the use of mobile apps to support clinical care was very positive [40]. Previous published systematic reviews showed that internet-based self-monitoring interventions and technologies had some moderately positive effects on maternal outcomes, such as levels of glycated hemoglobin A_1c_ and cesarean delivery rates, though discussion of psychosocial supports were seldom discussed [41-43].

Recent developments in digital health therapeutics include incorporating artificial intelligence, such as machine learning and natural language processing models. mHealth interventions, like wearable glucose monitoring devices, automated text messaging, and web-based health coaching have great opportunity to be integrated with these new advancements, which would allow for personalized health care support, though more work is required to understand the incorporation of machine learning into health care [44,45]. However, there are already existing applications for DM monitoring and management, in addition to broader digital health coaching applications such as the ProHealth eCoach prototype app by Chatterjee et al [46], that incorporate artificial intelligence, suggesting its potential for improving individual quality of life [47-50].

### Future Directions

Prioritization of user-centered design (UCD) that incorporates patient and provider perspectives in the development of DM-related mHealth technologies, especially among minoritized populations where mHealth may provide an avenue to bridge racial and ethnic health gaps, is of principal importance for health equity and cultural sensitivity [51-53]. UCD in mHealth interventions will allow DM support technologies to best promote timely health care engagement, intervention, and behavioral change in manners most acceptable to users. Qualitative data collection with mHealth stakeholders, such as pregnant people with DM and their health care providers, along with quantitative usability assessments, would allow for iterative feedback to improve mHealth implementation [5,16,17,54]. Incorporating an option to have a “social network” feature to add friends or family as a GDM mobile app used in a Nepalese hospital did, if desired, could potentially enhance individual sense of support through navigating DM in pregnancy [55]. Should an app like SweetMama enter the consumer space, UCD should play a predominant role in enabling effective and consistent user engagement. Moving forward, the SweetMama development team aims to incorporate missing perspectives from non–English-speaking patients and operationalize UCD principles to expand app accessibility, improve the user experience, and incorporate greater consideration of language diversity and the cultural preferences of users [56]. Our early-phase study of SweetMama contributes to the broader literature that demonstrates the promise of mHealth interventions for patients with DM during pregnancy, with potential for providing them with a greater sense of self-efficacy, community, and agency to preserve their physical and psychosocial health during pregnancy and in the long term.

## Abbreviations

DM: diabetes mellitus
GDM: gestational diabetes mellitus
mHealth: mobile health
MNT: medical nutrition therapy
T2DM: type 2 diabetes mellitus
UCD: user-centered design

## Data Availability

Given the sensitive and individualized nature of qualitative data, these data will not be publicly available. Investigators wishing to access these data may contact the corresponding author; data may be made available upon reasonable request and with evidence of appropriate approvals.

## References

[1] NA (2014). Diagnostic criteria and classification of hyperglycaemia first detected in pregnancy: a world health organization guideline. Diabetes Res Clin Pract.

[2] Alexopoulos AS, Blair R, Peters AL (2019). Management of preexisting diabetes in pregnancy: a review. JAMA.

[3] Yee LM, McGuire JM, Taylor SM, Niznik CM, Simon MA (2015). "I Was Tired of All the Sticking and Poking": identifying barriers to diabetes self-care among low-income pregnant women. J Health Care Poor Underserved.

[4] Yee LM, McGuire JM, Taylor SM, Niznik CM, Simon MA (2016). Social and environmental barriers to nutrition therapy for diabetes management among underserved pregnant women: a qualitative analysis. J Nutr Educ Behav.

[5] Yee LM, Leziak K, Jackson J, Niznik CM, Simon MA (2020). Health care providers' perspectives on barriers and facilitators to care for low-income pregnant women with diabetes. Diabetes Spectr.

[6] Young-Hyman D, de Groot M, Hill-Briggs F, Gonzalez JS, Hood K, Peyrot M (2016). Psychosocial care for people with diabetes: a position statement of the American diabetes association. Diabetes Care.

[7] Hinkle SN, Buck Louis GM, Rawal S, Zhu Y, Albert PS, Zhang C (2016). A longitudinal study of depression and gestational diabetes in pregnancy and the postpartum period. Diabetologia.

[8] Yu CY, Hung CH, Wang YY (2022). The impact of prenatal depression and diabetes management self-efficacy on postpartum stress and depression in women with gestational diabetes mellitus. J Clin Nurs.

[9] Muhwava LS, Murphy K, Zarowsky C, Levitt N (2020). Perspectives on the psychological and emotional burden of having gestational diabetes amongst low-income women in Cape Town, South Africa. BMC Womens Health.

[10] Craig L, Sims R, Glasziou P, Thomas R (2020). Women's experiences of a diagnosis of gestational diabetes mellitus: a systematic review. BMC Pregnancy Childbirth.

[11] Persson M, Winkvist A, Mogren I (2010). 'From stun to gradual balance'--women's experiences of living with gestational diabetes mellitus. Scand J Caring Sci.

[12] Tierney M, O'Dea A, Danyliv A, Noctor E, McGuire B, Glynn L, Al-Imari H, Dunne F (2015). Factors influencing lifestyle behaviours during and after a gestational diabetes mellitus pregnancy. Health Psychology and Behavioral Medicine.

[13] Sushko K, Strachan P, Butt M, Nerenberg KA, Sherifali D (2023). Understanding the self-management experiences and support needs during pregnancy among women with pre-existing diabetes: a qualitative descriptive study. BMC Pregnancy Childbirth.

[14] Tschirhart H, Landeen J, Yost J, Nerenberg KA, Sherifali D (2024). Perceptions of diabetes distress during pregnancy in women with type 1 and type 2 diabetes: a qualitative interpretive description study. BMC Pregnancy Childbirth.

[15] Fleming GA, Petrie JR, Bergenstal RM, Holl RW, Peters AL, Heinemann L (2020). Diabetes digital app technology: benefits, challenges, and recommendations. A consensus report by the European Association for the Study of Diabetes (EASD) and the American diabetes association (ADA) diabetes technology working group. Diabetologia.

[16] Yee LM, Leziak K, Jackson J, Niznik C, Saber R, Yeh C, Simon MA (2023). SweetMama: usability assessment of a novel mobile application among low-income pregnant people to assist with diabetes management and support. Diabetes Spectr.

[17] Yee LM, Leziak K, Jackson J, Strohbach A, Saber R, Niznik CM, Simon MA (2021). Patient and provider perspectives on a novel mobile health intervention for low-income pregnant women with gestational or type 2 diabetes mellitus. J Diabetes Sci Technol.

[18] Steinberg JR, Yeh C, Jackson J, Saber R, Niznik CM, Leziak K, Yee LM (2022). Optimizing rngagement in an mHealth intervention for diabetes support during pregnancy: the role of baseline patient health and behavioral characteristics. J Diabetes Sci Technol.

[19] Yee L, Taylor S, Young M, Williams M, Niznik C, Simon M (2020). Evaluation of a text messaging intervention to support self-management of diabetes during pregnancy among low-income, minority women: qualitative study. JMIR Diabetes.

[20] O'Connor S, Wang Y, Cooke S, Ali A, Kennedy S, Lee JJ, Booth RG (2023). Designing and delivering digital learning (e-Learning) interventions in nursing and midwifery education: a systematic review of theories. Nurse Educ Pract.

[21] Glaser B, Strauss A (2017). Discovery of Grounded Theory: Strategies for Qualitative Research.

[22] Hallberg LR (2006). The "core category" of grounded theory: making constant comparisons. International Journal of Qualitative Studies on Health and Well-being.

[23] Smith GN, Louis JM, Saade GR (2019). Pregnancy and the postpartum period as an opportunity for cardiovascular risk identification and management. Obstet Gynecol.

[24] Karavasileiadou S, Almegwely W, Alanazi A, Alyami H, Chatzimichailidou S (2022). Self-management and self-efficacy of women with gestational diabetes mellitus: a systematic review. Glob Health Action.

[25] Fareed N, Swoboda C, Singh P, Boettcher E, Wang Y, Venkatesh K, Strouse R (2023). Developing and testing an integrated patient mHealth and provider dashboard application system for type 2 diabetes management among medicaid-enrolled pregnant individuals based on a user-centered approach: mixed-methods study. Digit Health.

[26] Fischer S, Morales-Suárez-Varela M (2023). The bidirectional relationship between gestational diabetes and depression in pregnant women: a systematic search and review. Healthcare (Basel).

[27] Al-Hashmi I, Hodge F, Nandy K, Thomas E, Brecht ML (2018). The effect of a self-efficacy-enhancing intervention on perceived self-efficacy and actual adherence to healthy behaviours among women with gestational diabetes mellitus. Sultan Qaboos Univ Med J.

[28] Sayakhot P, Carolan-Olah M, Steele C (2016). Use of a web-based educational intervention to improve knowledge of healthy diet and lifestyle in women with gestational diabetes mellitus compared to standard clinic-based education. BMC Pregnancy Childbirth.

[29] Delanerolle G, Phiri P, Zeng Y, Marston K, Tempest N, Busuulwa P, Shetty A, Goodison W, Muniraman H, Duffy G, Elliot K, Maclean A, Majumder K, Hirsch M, Rathod S, Raymont V, Shi JQ, Hapangama DK (2021). A systematic review and meta-analysis of gestational diabetes mellitus and mental health among BAME populations. EClinicalMedicine.

[30] Huang S, Magny-Normilus C, McMahon E, Whittemore R (2022). Systematic review of lifestyle interventions for gestational diabetes mellitus in pregnancy and the postpartum period. J Obstet Gynecol Neonatal Nurs.

[31] McGovern L, O'Toole L, Houshialsadat Z, O'Reilly SL (2024). Women's perspectives on mHealth behavior change interventions for the management of overweight, obesity, or gestational diabetes: a qualitative meta-synthesis. Obes Rev.

[32] Skar JB, Garnweidner-Holme LM, Lukasse M, Terragni L (2018). Women's experiences with using a smartphone app (the Pregnant+ app) to manage gestational diabetes mellitus in a randomised controlled trial. Midwifery.

[33] Peleg M, Shahar Y, Quaglini S, Broens T, Budasu R, Fung N, Fux A, García-Sáez G, Goldstein A, González-Ferrer A, Hermens H, Hernando ME, Jones V, Klebanov G, Klimov D, Knoppel D, Larburu N, Marcos C, Martínez-Sarriegui I, Napolitano C, Pallàs À, Palomares A, Parimbelli E, Pons B, Rigla M, Sacchi L, Shalom E, Soffer P, van Schooten B (2017). Assessment of a personalized and distributed patient guidance system. Int J Med Inform.

[34] Borgen I, Garnweidner-Holme LM, Jacobsen AF, Bjerkan K, Fayyad S, Joranger P, Lilleengen AM, Mosdøl A, Noll J, Småstuen MC, Terragni L, Torheim LE, Lukasse M (2017). Smartphone application for women with gestational diabetes mellitus: a study protocol for a multicentre randomised controlled trial. BMJ Open.

[35] Kennelly MA, Ainscough K, Lindsay K, Gibney E, Mc Carthy M, McAuliffe FM (2016). Pregnancy, exercise and nutrition research study with smart phone app support (Pears): study protocol of a randomized controlled trial. Contemp Clin Trials.

[36] Chen Q, Carbone ET (2017). Functionality, implementation, impact, and the role of health literacy in mobile phone apps for gestational diabetes: scoping review. JMIR Diabetes.

[37] Kytö M, Koivusalo S, Ruonala A, Strömberg L, Tuomonen H, Heinonen S, Jacucci G (2022). Behavior change app for self-management of gestational diabetes: design and evaluation of desirable features. JMIR Hum Factors.

[38] Duan B, Liu Z, Liu W, Gou B (2022). Assessing the views and needs of people at high risk of gestational diabetes mellitus for the development of mobile health apps: descriptive qualitative study. JMIR Form Res.

[39] Tumminia A, Vitacolonna E, Sciacca L, Dodesini AR, Festa C, Lencioni C, Marcone T, Pintaudi B, Scavini M, Succurro E, Torlone E, Napoli A (2019). "MySweetGestation": a novel smartphone application for women with or at risk of diabetes during pregnancy. Diabetes Res Clin Pract.

[40] Smyth S, Curtin E, Tully E, Molphy Z, Breathnach F (2022). Smartphone apps for surveillance of gestational diabetes: scoping review. JMIR Diabetes.

[41] Lau Y, Htun TP, Wong SN, Tam WSW, Klainin-Yobas P (2016). Efficacy of internet-based self-monitoring interventions on maternal and neonatal outcomes in perinatal diabetic women: a systematic review and meta-analysis. J Med Internet Res.

[42] Garg N, Arunan SK, Arora S, Kaur K (2022). Application of mobile technology for disease and treatment monitoring of gestational diabetes mellitus among pregnant women: a systematic review. J Diabetes Sci Technol.

[43] Eberle C, Loehnert M, Stichling S (2021). Clinical effectiveness of different technologies for diabetes in pregnancy: systematic literature review. J Med Internet Res.

[44] Shan R, Sarkar S, Martin SS (2019). Digital health technology and mobile devices for the management of diabetes mellitus: state of the art. Diabetologia.

[45] Harrer S (2023). Attention is not all you need: the complicated case of ethically using large language models in healthcare and medicine. EBioMedicine.

[46] Chatterjee A, Prinz A, Gerdes M, Martinez S, Pahari N, Meena YK (2022). ProHealth eCoach: user-centered design and development of an eCoach app to promote healthy lifestyle with personalized activity recommendations. BMC Health Serv Res.

[47] Contreras I, Vehi J (2018). Artificial intelligence for diabetes management and decision support: literature review. J Med Internet Res.

[48] Dankwa-Mullan I, Rivo M, Sepulveda M, Park Y, Snowdon J, Rhee K (2019). Transforming diabetes care through artificial intelligence: the future is here. Popul Health Manag.

[49] Park SW, Kim G, Hwang YC, Lee WJ, Park H, Kim JH (2020). Validation of the effectiveness of a digital integrated healthcare platform utilizing an AI-based dietary management solution and a real-time continuous glucose monitoring system for diabetes management: a randomized controlled trial. BMC Med Inform Decis Mak.

[50] Graham SA, Pitter V, Hori JH, Stein N, Branch OH (2022). Weight loss in a digital app-based diabetes prevention program powered by artificial intelligence. Digit Health.

[51] LeRouge C, Wickramasinghe N (2013). A review of user-centered design for diabetes-related consumer health informatics technologies. J Diabetes Sci Technol.

[52] Brewer LC, Fortuna KL, Jones C, Walker R, Hayes SN, Patten CA, Cooper LA (2020). Back to the future: achieving health equity through health informatics and digital health. JMIR Mhealth Uhealth.

[53] Fareed N, Swoboda C, Wang Y, Strouse R, Hoseus J, Baker C, Joseph JJ, Venkatesh K (2023). An evidence-based framework for creating inclusive and personalized mHealth solutions-designing a solution for medicaid-eligible pregnant individuals with uncontrolled type 2 diabetes. JMIR Diabetes.

[54] Jackson J, Leziak K, Niznik CM, Yee LM (2021). Health care providers' utilization of and perspectives on mobile health technology for diabetes and pregnancy Support. Diabetes Spectr.

[55] Shanmugavel A, Shakya PR, Shrestha A, Nepal J, Shrestha A, Daneault JF, Rawal S (2024). Designing and developing a mobile app for management and treatment of gestational diabetes in Nepal: user-centered design study. JMIR Form Res.

[56] Birati Y, Yefet E, Perlitz Y, Shehadeh N, Spitzer S (2022). Cultural and digital health literacy appropriateness of app- and web-based systems designed for pregnant women with gestational diabetes mellitus: scoping review. J Med Internet Res.

